# Highlights in applied evolutionary biology

**DOI:** 10.1111/eva.12040

**Published:** 2012-12-24

We are excited to introduce this new section in Evolutionary Applications. *Highlights* will feature recently published or (available online) papers that use evolutionary biology to address questions of practical importance. Below we have summarized the abstracts of the papers and encourage you to read the full paper for more details. We welcome suggestions for papers to include in this section and we encourage you to use this section to keep up to date on the latest advances in applied evolutionary biology.

## Climate Change

Leal, M and A. R. Gunderson. 2012. Rapid Change in the Thermal Tolerance of a Tropical Lizard. The American Naturalist 180: 815–822.

The authors measured the lower and upper thermal tolerances of a lizard recently introduced to Miami, Florida from Puerto Rico. The introduced population was able to tolerate significantly colder temperatures than the source population, suggesting that changes in thermal tolerance occurred relatively rapidly (∼35 generations). The data suggest that the thermal physiology of tropical lizards is more labile than previously proposed.

Olson, M. S., N. Levsen, R. Y. Soolanayakanahally, R. D.Guy, W. R.Schroeder, S. R.Keller, and P. Tiffin. The adaptive potential of *Populus balsamifera* L. to phenology requirements in a warmer global climate. Molecular Ecology. doi: 10.1111/mec.12067

The authors use common garden and association mapping approaches to quantify genetic variance and identify important growth-related traits in *P. balsamifera*. The study demonstrates that abundant heritable genetic variation exists for phenological response to changes in the length of the growing season. The distribution of SNP genotypes also suggests that many geographic regions may harbour sufficient diversity in functional genes to facilitate adaption to future climatic conditions.

## Disease Biology

Atkins, K. E., A. F.Read, N. J. Savill, K. G. Renz, A. F. Islam, S. W. Walkden-Brown, and M. E. J. Woolhouse. Vaccination and reduced cohort duration can drive virulence evolution: Marek's disease virus and industrialized agriculture. Evolution. doi: 10.1111/j.1558-5646.2012.01803.x

Marek's disease virus (MDV), a commercially important disease of poultry, has become substantially more virulent over the last 60 years. In this paper the authors use virulence theory to show that both vaccination and reduced lifespan of poultry could have led to the increase in viral virulence. These results illustrate the dramatic impact anthropogenic change can potentially have on pathogen virulence.

Schneider, P., A. S. Bell, D. G. Sim, A. J. O'Donnell, S. Blanford, K. P. Paaijmans, A. F. Read, and S. E. Reece 2012. Virulence, drug sensitivity and transmission success in the rodent malaria Plasmodium chabaudi. Proc. R. Soc. B. 279: 4677–4685.

The authors test the hypothesis that virulent malaria parasites are less susceptible to drug treatment than less virulent parasites. In all treatments, virulent parasites were less sensitive to pyrimethamine and artemisinin, the two antimalarial drugs we tested. Virulent parasites also achieved disproportionately greater transmission when exposed to pyrimethamine. Overall, the data suggest that drug treatment can select for more virulent parasites. The authors propose that drugs targeting transmission stages (such as artemisinin) may minimize the evolutionary advantage of virulence in drug-treated infections.

Ujvari B., A.-M. Pearse, S. Peck, C. Harmsen, R. Taylor, S. Pyecroft, T. Madsen, A. T. Papenfuss, K. Belov. Evolution of a contagious cancer: epigenetic variation in Devil Facial Tumour Disease. Proc R Soc B. http://dx.doi.org/10.1098/rspb.2012.1720

Devil Facial Tumour Disease (DFTD) is a highly contagious cancer and is driving Tasmanian devils (*Sarcophilus harrisii*) to extinction. The authors explore the Devil Facial Tumour (DFT) epigenome and the genes involved in DNA methylation homeostasis. Their findings demonstrate that DFTD should not be treated as a static entity, but rather as an evolving parasite with epigenetic plasticity. The authors suggest that understanding the role of epimutations in the evolution of this parasitic cancer will provide unique insights into the role of epigenetic plasticity in cancer evolution and progression in traditional cancers that arise and die with their hosts.

## Domestication

Koziol, L., L. H. Rieseberg, N. Kane, and J. D. Bever. Reduced drought tolerance during domestication and the evolution of weediness results from tolerance-growth trade-offs. Evolution. doi: 10.1111/j.1558-5646.2012.01718.x

To investigate whether changes in resource allocation occurred during domestication or the evolution of weediness, the authors compared the mycorrhizal responsiveness, growth, and drought tolerance of nine native ruderal, nine agriculturally weedy (four U.S. weedy and five Australian weedy), and 14 domesticated populations (eight ancient landraces and six improved cultivars) of the common sunflower (*Helianthus annuus)*. Overall, the authors found that trade-offs between drought tolerance and several aspects of plant growth, including growth rate, allocation to flowering, and root architecture, govern evolution during sunflower domestication and the invasion of disturbed habitat.

## Invasion biology

Clark, L. V. and M. Jasieniuk. 2012. Spontaneous hybrids between native and exotic *Rubus* in the Western United States produce offspring both by apomixes and by sexual reproduction. Heredity 109: 320–328

Many invasive species may adapt to new environments via sexual recombination during range expansion, while at the same time having the benefits of asexuality. The authors hypothesize that new invasive lineages of *Rubus* could arise from hybridization between the native *Rubus*, which reproduces sexually and the exotic naturalized *Rubus*, which reproduces by pseudogamous apomixis. Using microsatellite and chloroplast markers, the authors show that some hybrids have arisen, and this system models the early stages of evolution of new invasive lineages. Mixed reproductive systems such as those described here may be an important step in the evolution of asexual invasive species.

## Resource conservation and management

Richard, A., M. Dionne, J. Wang, and L. Bernatchez. Does catch and release affect the mating system and individual reproductive success of wild Atlantic salmon (Salmo salar L.). Molecular Ecology. doi: 10.1111/mec.12102

The authors document the breeding system of a wild population of Atlantic salmon (*Salmo salar* L.) by genetically sampling every returning adult and assessed the determinants of individual fitness. They then quantified the impacts of catch and release fishing (C&R) on mating and reproductive success. The impact of C&R on the number of offspring was size dependent, as the reproductive success of larger fish was more impaired than smaller ones. This study improves our understanding of the complex reproductive biology of the Atlantic salmon and is the first to investigate the impact of C&R on reproductive success. This study expands the management toolbox of appropriate C&R practices that promote conservation of salmon populations and limit negative impacts on mating and reproductive success.

